# Affordances and the musically extended mind

**DOI:** 10.3389/fpsyg.2013.01003

**Published:** 2014-01-06

**Authors:** Joel Krueger

**Affiliations:** Department of Sociology, Philosophy, and Anthropology, University of Exeter AmoryExeter, UK

**Keywords:** music, affordances, extended cognition, emotions, emotion regulation, phenomenology

## Abstract

I defend a model of the musically extended mind. I consider how acts of “musicking” grant access to novel emotional experiences otherwise inaccessible. First, I discuss the idea of “musical affordances” and specify both what musical affordances are and how they invite different forms of entrainment. Next, I argue that musical affordances – via soliciting different forms of entrainment – enhance the functionality of various endogenous, emotion-granting regulative processes, drawing novel experiences out of us with an expanded complexity and phenomenal character. I argue that music therefore ought to be thought of as part of the vehicle needed to realize these emotional experiences. I appeal to different sources of empirical work to develop this idea.

## INTRODUCTION

One of the main reasons we listen to music is to regulate our emotions (Krumhansl, 2002). Musical activity – temporally patterned activity by which individuals or groups produce or perceive intentionally structured sound (Cross, 2001; Croom, 2012, p. 2) – is often undertaken in the service of emotion regulation. We listen to music to elicit powerful feelings.^1^ And by eliciting powerful feelings, music can function as a tool for motivating both individual and collective actions. Considerations such as these led Small (1998) to coin the term “musicking” to stress the active character of our musical engagements. Following Small, I will use “musicking” to encompass the different ways that we actively engage with – and indeed *use* – music to animate behavior, cultivate and refine affective experiences, and orient ourselves to others and the world more generally.^2^

I specifically consider how musicking-in-*listening* enables us to develop novel emotions and, in so doing, expand our emotional repertoire. I argue that music does, indeed, often grant access to novel emotions – and it does so by scaffolding and enhancing the functional complexity of certain endogenous resources, thus granting phenomenal access to experiences that we would be otherwise unable to develop. First, I discuss the idea of “musical affordances” and specify both what musical affordances are and how they invite different forms of musical engagement. Next, I argue that musical affordances enhance the functional complexity of various endogenous, emotion-granting regulative processes, drawing novel experiences out of us with an expanded complexity and phenomenal character. I appeal to different sources of empirical work to develop this idea. In so doing, I suggest that, since music is an essential resource needed to access these experiences, it is therefore warranted to speak of the musically extended mind.

## MUSICKING AND MUSICAL AFFORDANCES

In everyday life, we do many different things with music. A common use of music is as an atmosphere-enriching sonic additive. Dynamic beat-heavy music can elevate spirits at a party, creating a joyous atmosphere, and compelling listeners to mingle and dance; slower reverent music – at a funeral, for instance – can have the opposite effect, bringing about an atmosphere of quiet grief and remembrance.^3^ Music can also coordinate actions both solitary and social. In elevating our mood and sharpening our attentional focus (Sridharan et al., 2007), music can get us into different states of action-readiness necessary for various activities, including combat (Gittoes, 2004; Protevi, 2010), athletics (DeNora, 1986), erotic encounters (DeNora, 1997), dining out (Caldwell and Hibbert, 1999), or studying (Rauscher et al., 1993).

The point of these observations is that within everyday life, music is generally not perceived merely as an esthetic object for passive contemplation. Rather, we perceive it as a resource we can *use* to do different things, much the same way we perceive tools and technologies as resources that help us accomplish different tasks. Music, I suggest, is experienced as having *instrumental* value. And what I suggest further is that musical affordances are what specify the different sorts of things we can do with music. As we will see, we are attuned and responsive to musical affordances from birth – and probably even earlier.^4^

I am appropriating the term “affordances” from ecological psychology, particularly the work of Gibson (1979). There is much debate over what Gibson meant with this term, precisely, and how best to understand it. For the purposes of this paper, we can leave this debate to others (see, for example, Heft, 2001; Chemero, 2003). When I speak of affordances in what follows, I mean simply action possibilities in a perceiver’s environment that are specified *relationally*, that is, both by (1) particular structural features of the environment and things in it, as well as (2) the repertoire of sensorimotor capacities the perceiver employs to detect and respond to these structural features. A perceiver, in virtue of being embodied in a particular sort of way – and possessing an accumulated history of environmental interactions – will experience affordances as furnishing different sets of interactive possibilities (cf. Shaw and Turvey, 1981; Heft, 1989; Chemero, 2003).

For an adult human, for example, a chair (in virtue of its structural properties such as height, shape, texture, etc.) affords sitting, standing on, or picking up; for an infant or a cat or a lizard, it affords none of these things – but it does afford crawling or hiding under. Different perceivers who vary in their biological, physiological, morphological, and kinematic details – as well as their goal-directed activities (Mazet, 1991) and individual histories – will thus detect and respond to different affordances. The same chair will exhibit perceiver-relative “activity signatures”: functional properties that can be expressed as “for + verb” (e.g., a chair is *for* sitting or *for* hiding under; cf. Beck, 1987). Accordingly, for an ecological, affordance-based approach to perception, what matters “is not merely the world in its objective qualities, but the world as perceived by organisms” (Reybrouck, 2012, p. 394).

This relational way of thinking about how affordances are specified is beneficial. It helps to clarify how the same objective environment can furnish different meanings for different perceivers. Again, this is because the experience of particular environmental affordances as interactively salient will co-vary with the details of that perceiver’s history and embodiment. As Gibson (1979, p. 8) puts it, there is a mutuality between perceiver and environment.

In what follows, I am mainly concerned with the perceiver’s experience of affordances – the phenomenology of their sensorimotor responsiveness to affordances.^5^ I am particularly interested in the way that musical affordances exhibit a kind of *felt allure* (cf. Rietveld, 2008). This idea refers to the way that we often experience music as affectively irresistible; we are drawn to it, emotionally – often in a very powerful way – in part because we immediately recognize it as *meaningful*, that is, as something with a distinctive activity signature that we can use or do things with (cf. Krueger, 2011a; Reybrouck, 2012). Part of music’s impact comes via the way that it presents itself as an environmental resource inviting interaction (both potential and actualized), from simple affective and behavioral reactivity to more complex, synchronized forms of engagement. The first personal character of this affective allure thus consists in the manner that musical affordances are, from a very early age, experienced as *potentiating* a delimited range of context-sensitive responses (Rietveld, 2008, p. 977). More on this idea as we proceed.

Considering auditory affordances is not a novel endeavor. Gibson (and those who follow him) speak mainly of the visual detection of affordances. But since affordances are specified via the perceptual pick-up of structured information in the environment, they are potentially available via any modality – including audition. And despite the optical emphasis of most discussions of affordances, some working within this tradition have investigated how the perception of auditory events specify different affordances (e.g., Warren and Verbrugge, 1984; Warren et al., 1987; Heine and Guski, 1991; Gaver, 1993a,b; Cummins, 2009). For example, Warren et al. (1987) found evidence for the intermodal character of auditory event perception: in ball-bouncing tasks, the information observers used to determine bounce height was identical in both visual and auditory conditions (see also Kohler et al., 2002). We will return to the intermodal character of auditory perception below.

Nevertheless, despite this existent work on ecological acoustics, the notion of “affordances” has not received much application in music cognition literature generally – although there are a few exceptions here, too (e.g., Gaver, 1993a,b; Reybrouck, 2001, 2005, 2012; Windsor, 2004; Clarke, 2005; Krueger, 2011a; Windsor and de Bézenac, 2012). As we will see, however, the notion is valuable within a musical context. It can help elucidate the extent to which audition (including music listening) and action are fundamentally interwoven (Warren et al., 1987). It also helps to clarify the informational richness of music (Parncutt, 2009) and the manner by which musical information exerts its characteristically strong pull on us (i.e., its affective allure) as we do things with it in everyday life (DeNora, 2000).

So what does music afford the listener?^6^ And what are the structural features of music, as perceived by the listener, that specify these affordances? An obvious but nevertheless important initial answer to the first question is that music affords *movement*. Unlike non-musical noise in our environment – the sound of a passing car outside our window, say, or a slamming door, the laughter of children playing in the yard, or the steady murmur of water flowing through a nearby brook – music generally presents a sonic profile that invites a synchronous motor response (Clayton et al., 2005; Bispham, 2006). This response can be as subtle as tapping our fingers or nodding our head; or it can be an elaborately choreographed dance routine. While a slamming door may solicit its own sort of motor response (e.g., a sudden grimace or flinch), that sound event does not invite synchronous and sustained motor *engagement*; it lacks the requisite sonic profile, the relevant affordances. But music often does offer such a profile (in a way to be clarified momentarily).

Music in this way affords what Windsor and de Bézenac (2012, p. 112) helpfully term “advancing behavior” : a mode of sustained and responsive engagement in which the acoustic structure of a musical event – features like cyclical patterns of rhythmic or melodic accentuation, goal-directed tonal movements, modulation of volume or intensity, etc. – draws the listener into a patterned response reflecting the dynamics of this acoustic structure. A simple example is the way that the buoyant melody of a children’s song seems to draw a similar range of bouncy, swaying movements of the head and trunk from those who listen to it. To a certain extent, the morphology of our advancing behavior mirrors the dynamics of the experienced music – although there are, of course, individual differences in the reactive behavior we display.

Music thus often affords responsive movements in a way most other environmental sounds do not. But we can specify more precisely the character of this musically solicited responsive movement. As the example of the children’s melody demonstrates, musical dynamics exert an *organizational control* over our motor responses. In other words, the acoustic structure of a music event affords a fine-grained synchronization or bodily alignment with the music. Again, a simple example is the way that we instinctively start tapping our fingers or nodding our heads along with the periodic modulations of melody or rhythm of a piece of music that catches our attention (cf. Repp, 2005). Even in these rudimentary cases of everyday listening, music exhibits a strong affective allure; it is difficult to ignore it or repress our bodily responses (Brown and Parsons, 2008), even very early in life (Haslbeck, 2004; Zentner and Eerola, 2010). From the start, we experience music as something that naturally invites this kind of synchronized interaction.

Music therefore affords not just movement but, crucially, *entrainment*. Entrainment is a concept from complex systems theory. It occurs when two or more independent oscillatory processes are synchronized with each other, gradually adjusting toward – and eventually locking into – a common phase and/or periodicity (Clayton et al., 2005; Will and Turow, 2011). A classic example of entrainment – first identified by the Dutch physicist Christiaan Huygens in 1665 – involves the movement of two pendulums gradually coming into phase-synchrony via minute vibrations in the walls and floor (Winfree, 2001; Bennett et al., 2002). The notion has since been applied to multiple domains including work in mathematics, as well as physical, biological, and social sciences. Entrainment has been described in multiple systems and at multiple time-scales: from Asian fireflies flashing in synchrony (Buck and Buck, 1968), human interactants synchronizing gestures, and speech patterns (Chartrand and Bargh, 1999), to a group gradually transitioning from random to synchronized clapping (Néda et al., 2000). Generally, entrainment emerges via mutually modulatory interaction between different oscillators (e.g., two swinging pendulums influencing one another’s movements). But the interaction can also be one-way, such as with human entrainment to diurnal cycles – or music.^7^

Music studies have thus far made little use of the notion of entrainment. This is surprising, as the idea seems to accord naturally with how we engage with music. It seems particularly well-suited to capture the impact that music and musical rhythms have on the reactive behavior of embodied listeners (Will and Turow, 2011, p. 12). Since music is a structured “sound-time phenomenon” (Reybrouck, 2012), rhythm becomes a key component for bodily marking the temporal development of a musical event. Musical entrainment occurs via the behavioral coordination resulting from an individual’s responsiveness to rhythmic signals, such as a simple (e.g., 1:1 in-phase or anti-phase) or more complex (e.g., 2:3 or 3:4) phase relations (Phillips-Silver et al., 2010; cf. Bispham, 2006; Merker et al., 2009; Nozaradan et al., 2012). This way of coordinating our reactive behavior to the music is a way of bodily gearing onto musical structures. This process emerges and takes shape as the music unfolds around us in acoustic space, where we (often unthinkingly) coordinate our movements with the dynamics of this unfolding – much the way that a dance between two partners emerges dynamically, in real-time, from the ongoing interplay and synchronization of each partner’s movements and their individual responses to what the other is doing. Temporality is thus a key feature of musical entrainment.^8^

But musical entrainment also has another key feature relevant to this discussion: *affective synchrony* (Phillips-Silver and Keller, 2012, p. 1; cf. Trevarthen and Malloch, 2002; Janata et al., 2012). This notion refers to the sharing of feeling states that often emerge when individuals entrain their movements with one another – for example, when jointly listening to or performing music (Keller and Appel, 2010), or when engaging in non-musical activities such as simply walking in sync with a partner, or having a conversation and (unconsciously) mimicking their postures, facial expressions, and gestures (Hatfield et al., 1993; Chartrand and Bargh, 1999; Lakin and Chartrand, 2003; Krueger and Michael, 2012). Within the context of musical entrainment, affective synchrony refers to the pleasure we take simply in moving our bodies in time with the music, letting musical rhythms (and the movements they solicit) draw certain felt responses out of us – and, when others are present, the pleasure we take in *sharing* this process (i.e., of getting into the “groove” together; Pressing, 2002; Madison, 2006; Janata et al., 2012; cf. Schutz, 1951). Affective synchrony in this way seems to be a central part of the affective allure of musicking. We engage with music because, unlike most other non-musical sounds, it affords synchronously organizing our reactive behavior and felt responses; and we take pleasure in letting music assume some of these organizational and regulative functions that, in other contexts, normally fall within the scope of our own endogenous capacities. In other words, we “offload” some of these regulative processes onto the music and let it do some of the work organizing our emotional responses for us.^9^

Via soliciting entrainment responses, music thus exerts an organizational control over the form and dynamics of our embodied musical engagements. This is reflected in the way that different styles of music invite different patterns of entrainment with their own unique temporal signature and affective synchrony. Entrainment responses that fit Tango or Salsa, for example, may differ significantly – as will those more organically aligned with the acoustic structure of different musical genres like Pop, Country, Electronic, or Post-Rock.^10^ The spatiotemporal movements of our entrainment responses to music are thus organized in a way that reflects the hierarchically organized rhythmic and melodic structure of the music (Windsor and de Bézenac, 2012, pp. 112–113; cf. Leman, 2008; Burger et al., 2013). The pitch intervals and contour of melody, for instance, establish trajectories through mental representations of pitch space that – at least potentially – map cross-modally onto movement possibilities within physical space (Phillips-Silver and Keller, 2012, p. 2; cf. Eitan and Granot, 2006; Rusconi et al., 2006; Lidji et al., 2007; Eitan and Timmers, 2010). Phenomenologically, we *feel* certain movements to be more contextually appropriate than others, relative to metrical and melodic patterning within musical structures. For example, while the triple meter of the waltz is not experienced as affording marching – trying to march to a waltz feels somehow odd, as though the music is working against us and our bodily gestures are not “fitting into” the appropriate musical cues – a duple meter at the correct tempo would establish a different entrainment context, one in which marching responses do feel more appropriate (Windsor and de Bézenac, 2012, p. 113). The acoustic structure of the music-as-heard thus determines the form of our musical advancing behavior; it shapes how we interact with and “inhabit” the music, experientially, and what we do with it (cf. Leman and Naveda, 2010; Naveda and Leman, 2010).^11^

So what is it about music that affords entrainment? I have considered this idea in some detail elsewhere (Krueger, 2011a). For our purposes, we can answer this question briefly. The salient structural features are musical features that contribute to its *dynamic* quality: its character as temporally extended, spatially and acoustically complex sound event exhibiting its own internal organizational coherence, its own compositional logic. The particular structural features that comprise musical dynamics are many, and quite varied: cyclical patterns of rhythmic or melodic accentuation, goal-directed tonal movements, modulation of volume, intensity, cadence, or tempo, etc. This is but a partial list; there are other features of music that potentially contribute to its dynamism. The point is that these qualities – and many more like them – are what imbue music with its “aliveness,” its vitality – and its ability to summon impressions of movement, expression, and spatiality (Molnar-Szakacs and Overy, 2006; cf. Krueger, 2011a,b). In other words, they are structural features of music that determine it to be an event that unfolds dynamically and coherently in acoustic space and time, and which – when paired with an appropriately sensitive listener – thus affords specific forms of interaction and motor entrainment. Again, we cannot entrain with non-musical sounds – or rather, at least not with the same degree of reactive *complexity* – precisely because they lack the dynamics and organizational coherence of music. Theirs is a sonic profile that does not specify well-articulated and, crucially, *varied* entrainment possibilities – that is, possibilities for *musicking*.^12^

## AFFORDANCES, MUSICKING, AND THE EXTENDED MIND

I now want to consider the way that music and musical affordances grant access to kinds of experiences otherwise inaccessible. The specific idea I want to defend is this: at times, music serves as an external (i.e., outside-the-head) resource that can profoundly augment, and ultimately *extend*, certain endogenous capacities. When we engage in bouts of musicking, we potentially use music to become part of an integrated brain–body–music system – and within this extended system, musical affordances provide resources and feedback that loop back onto us and, in so doing, enhance the functional complexity of various motor, attentional, and regulative capacities responsible for generating and sustaining emotional experience. It is thus sensible to speak of the musically extended (emotional) mind.

To head off an immediate worry, music does not necessarily express or represent neatly articulated emotions; perhaps it does not represent emotions *at all*.^13^ Whether or not music can be said to properly represent emotions is a heated debate; it need not concern us here (see, e.g., Kivy, 1989; Robinson, 2005; Zangwill, 2007). Rather, following Sloboda, I instead affirm the idea that “the so-called power of music may very well be in its emotional cue-impoverishment. It is a kind of emotional Rorschach blot” (Sloboda, 2000, p. 26). On the affordance-based conception of music I am here advocating, music is conceived of as an information-rich perceptual object. But representations of emotions need not be part of its informational structure. Rather, what matters is that music affords a sonic profile enabling the listener to *use* it to cultivate and refine specific emotional experiences. Music, when being used by the engaged listener, therefore becomes part of the extended vehicle by which these experiences are realized.

Framed thusly, I am intentionally situating this affordance-based approach to music listening within discussions of the extended mind thesis (Clark and Chalmers, 1998; Clark, 2008; cf. Chemero, 2009; Menary, 2010), or the *hypothesis of extended cognition* (HEC), as it is sometimes called. According to HEC, the vehicles of cognition need not be confined to the head. Within certain circumstances, artifacts, tools, technologies, cultural institutions – and perhaps even other people – can become part of a spatially extended cognitive system in virtue of the active role they play in driving various cognitive processes such as reasoning, remembering, planning, calculating, perceiving, and navigating our environment, etc.

One way to think about HEC is therefore to understand it as a theory of *access*. Without the ongoing, active contribution of external resources, we cannot access the different cognitive functions they support. For example, certain mathematical calculations are only accessible with the aid of external props such as a pen and paper, calculator, or computer; similarly, storage devices (books, maps, cloud computing services, stories and cultural narratives, sticky notes on computer monitors) give us access to short- and long-term recall that far exceeds our native biological capacities (Clark, 2003). When we integrate our biological capacities with these external resources, we thus extend – in a very literal sense – our endogenous function and become part of an increasingly complex, functionally and spatially expanded cognitive system (cf. Sutton, 2006; Wilson and Clark, 2009). We access new and more powerful forms of cognition.

Discussions of HEC have only recently begun to move beyond a consideration of the lone agent fiddling with different bits of his material environment to consider the possibility of both interpersonally and emotionally extended cognition (see, e.g., Gallagher, 2013; Krueger, submitted; Slaby, forthcoming; Stephan et al., 2014). This expanded perspective on HEC is a welcome development. Cognition rarely (if ever) proceeds independently of emotions and affect; and it is, moreover, always situated in encompassing social and cultural ecosystems, comprised of constantly evolving cultural practices, that profoundly shape its manner of functioning (Hutchins, 2008). Considering music as a rich affective and sociocultural resource can help further motivate this new orientation.

### MUSICKING AS EXTERNALLY DRIVEN REGULATION

I have suggested that acting upon musical affordances can potentially enhance the functional complexity of various motor, attentional, and regulative capacities responsible for generating and sustaining emotional experience. It should be noted that, while we can mark a conceptual distinction between these three dimensions of musicking, these are nevertheless artificial distinctions. In real-world musicking, these dimensions are interrelated: the motor dimension of music listening impacts attention – spontaneously nodding along to a musical rhythm, for instance, is a kind of motorically motivated attending to particular sound elements – and both are ultimately modulated by music’s external regulatory force. So, while I occasionally focus on individual dimensions of musicking in isolation, I will in what follows also try to highlight their interrelation.

We can begin by reemphasizing the extent to which musical experiences are enactive, multimodal processes involving the integration of perception and action (Molnar-Szakacs and Overy, 2006; Krueger, 2009, 2011b). This is not just the case for musical *performance*. Clearly when performing music, the different sensorimotor strategies we use to engage with the instrument are enhanced by the affordances unique to that instrument; there are certain movements and acts of sound creation unique to a guitar, trumpet, or xylophone. Within the context of musical performance, agent and instrument form a coupled system in which morphologies of movement and musical structure are mutually constraining (cf. Baily, 1992; Davidson, 2012; Thompson and Luck, 2012).

But a similar principle also applies to music *listening*. We have already observed how readily music draws movement out of us: from simply tapping our fingers to participating in an elaborately choreographed dance or music-fueled religious ritual, musical experience is multimodal (Iyer, 2002; Janata et al., 2012). And by soliciting different forms of movement, music functions as a real-time emotion regulator. It solicits – and crucially, modulates the general shape of – attention and expressive behavior responsive for generating specific affective experiences (cf. Damasio, 1994; Holstege et al., 1996; Niedenthal, 2007).

Consider first how we use music to facilitate activities like exercise or manual labor (Karageorghis and Terry, 1997; Robinson, 2005, pp. 395–405). Music is known to sharpen and sustain our attentional focus (Sridharan et al., 2007), which can be helpful when working through strenuous activity. More strikingly, however, music can also reduce how *difficult* strenuous activity feels. It does so not simply by masking unpleasant proprioceptive feedback one gets during strenuous exertions – that is, by directing our attention away from unpleasant sensations – or by providing a calming effect that reduces muscle tension and increases oxygenation (although these consequences are likely part of its efficacy). Additionally, and more pertinent to this discussion, music can function as a kind of surrogate agency: a “musical agency” (Fritz et al., 2013). It directly modulates reactive behavior and affect (“emotional motor control”) on the listener’s behalf by providing ongoing feedback in the form of “virtual goals”: dynamic representations of movement-possibilities “with anticipatable but adaptable endpoints” that enable listeners to “regulate and monitor the extent and the timing of their movements more effectively” (Fritz et al., 2013). In short, music assumes an external regulative function. Rhythmic structures, in particular, keep time for the listener and contour the shape of musically induced bodily expressions and movement patterns (i.e., via rhythmic accentuations, increased or decreased tempo, etc.) that propel them through their strenuous activity. In these cases, the listener offloads certain regulative functions onto the music; instead of attending to this time-keeping, action-monitoring information herself, she allows the music to tell her both *when* and *how* to act. Along with whatever additional positive physiological effects music may have, then, this offloading reduces the listener’s cognitive burden and, predictably, lowers her feeling of perceived exertion since she now has one less thing to worry about and can thus direct her attention elsewhere. As a point of contrast, consider how dramatically one’s mood and behavior is altered – and how *difficult* the strenuous activity suddenly becomes – when this offloading possibility is suddenly unavailable: for example, when one’s MP3 player abruptly shuts off in the middle of a workout due to a dead battery (cf. Lim et al., 2009).

Looking at concrete instances such as this of how we use music in everyday life reinforces how deeply movement and music listening are interrelated. But to further understand the motoric dimension of musicking, we must note that music does not merely *cause* us to move and feel in particular ways. The causal chain does not simply proceed in a tidy linear fashion from music (as heard) to action (as caused by heard music). It also runs the other way. The experience of agency is intrinsic to how we hear and respond to music. In other words, as the case discussed above suggests, there is an irreducibly *interactive* dimension to music perception – even when “passively” listening.

This idea is confirmed by other empirical studies. In one of them, Chen et al. (2008) found that subjects who listened to musical rhythms with the knowledge that they would soon be asked to tap along with these rhythms showed activity in the supplementary motor area, mid-premotor cortex, and cerebellum. However, “naïve” subjects – subjects unaware that they, too, would soon be asked to tap along – displayed the same activity; passive listening (i.e., listening without anticipation) recruited the same motor regions. It seems that, even for listeners not overtly engaged in behaviorally responding to music, musical affordances were nevertheless perceived as soliciting engagement. Other studies likewise support the idea that the motor system is involved in processing musical rhythms (see, e.g., Sakai et al., 1999; Grahn and Brett, 2007; cf. Kohler et al., 2002). They also suggests that the experience of agency and bodily movement plays a role in shaping the perceptual character of music-as-heard – even, once again, during supposedly passive listening episodes (Phillips-Silver and Trainor, 2005).

These studies, and others like them, suggest that we quite literally hear music through movement. Perceived motor potentialities are a central feature of our musical “advancing behavior” (Windsor and de Bézenac, 2012); they frame how music is brought to phenomenal presence *as* music. It is telling to observe how the phenomenology of musicking is altered when motor potentialities are no longer a part of the listening experience. In cases of congenital amusia (Ayotte et al., 2002) – the inability to recognize musical melody, time-changes, or discriminate pitch despite otherwise normal hearing – music is experienced as presenting a very different sort of sonic profile. Total amusiacs report that they experience music as having a highly disagreeable character: it may sound like a car screeching or the banging of pots and pans (Sacks, 2007, pp. 98–119). Accordingly, the phenomenal character of their musicking experience is very different from that of the non-amusiac in that they fail to detect musical affordances; they are not attuned to music’s affective allure and thus do not experience it as potentiating a range of entrainment responses. This lack of responsiveness is born out in studies showing that amusiacs have great difficulty synchronizing bodily movements with music, despite an otherwise normal ability to synchronize with sequences of non-musical sounds (Dalla Bella and Peretz, 2003; cf. Krueger, 2009, pp. 118–121).

We thus hear music *qua* music through the motor potentialities it affords. But again, to return to an earlier point, music clearly also *solicits* movement, different forms of entrainment (both voluntary and involuntary) that shape what we hear, how we hear it, and how we respond emotionally. For example, listeners in one study exhibited spontaneous facial mimicry when presented with auditory-visual presentations of emotional singing (Chan et al., 2013). Happy singing elicited happy facial expressions, sad singing elicited sad expressions. But this effect does not rely upon the observation of another person. More strikingly, spontaneous facial expressions were also observed in individuals listening to expressive non-vocal music (Witvliet and Vrana, 1996; Lundqvist et al., 2009). These motor solicitations appear to occur from our earliest exposure to music. Infants discriminate musical from non-musical sounds: they coordinate their reactive behavior to the former but not the latter (Trainor and Heinmiller, 1998; Zentner and Kagan, 1998; Nawrot, 2003). Even neonates and preterm infants bodily entrain with sung lullabies and consonant music, syncing respiratory patterns, sucking (both rhythm and intensity), tongue and mouth protrusions, eye opening and closing, and vocalizations along with the rising and falling of melodic contour (Haslbeck, 2004; cf. Krueger, 2013a). This entrainment has cognitive and emotional significance. It leads to greater equilibrium between endogenous and exogenous processes, buttressing the infant’s attentional and behavioral organization and promoting stabilization of affect (DeNora, 2000, p. 79).

For our purposes, there are two important points here. First, our engagement with music is always *reciprocal* and *interactive*. Even when “passively” listening, we are, in fact, not really passive listeners. Rather, we are active perceivers: we latch onto musical affordances and respond, motorically, to the solicitations of these affordances – even if this response is at times involuntary. And crucially, the way we latch onto musical affordances determines the phenomenal shape of how the music comes back to us, so to speak, how the music is constituted, perceptually (cf. Phillips-Silver and Trainor, 2005). Again, recall how dramatically the phenomenal shape of music changes when motor potentialities are absent (e.g., as with amusia). The amusiac and non-amusiac may be said to listen to the same piece of music considered purely as a sonic object. But what they *hear* in the music and what they *get* out of it will differ greatly. For amusiacs, music is perceptually encountered as a sonically impenetrable *object*; for non-amusiacs, it is perceptually encountered as a structured acoustic *landscape* affording various forms of reactive behavior. Motor potentialities thus partially constitute the perceptual character of the music-as-heard. And via motor entrainment, we can be said to *integrate* with musical affordances in a dynamic, two-way relation of continuous reciprocal causation (Clark, 1997, p. 165): what we hear determines how we respond, which shapes what we hear, which informs our further responses, etc. Motor entrainment is one of the mechanisms that secures this ongoing, mutually modulatory integration.^14^

Second, these studies also support the idea that acting with musical affordances potentially enhances the functionality of various micro-practices responsible for emotional experience and self-regulation. Via the iterative cycles of motor entrainment that emerge within the music-listener system, ongoing feedback/feedforward loops are generated that have regulatory significance at both the neural and behavioral level. Without the ongoing input from these musical affordances, however, these regulatory processes would not emerge – much the same way we cannot access certain cognitive functions (e.g., working out long multiplication problems) in the absence of ongoing feedback from the relevant external props (e.g., pen and paper; cf. McClelland and Rumelhart, 1986). Musical dynamics thus provide external scaffolding supporting the synchronic emergence of novel music-specific experiences.

At the neural level, the perception of musical gestures – rhythmic structures and melodic contour, which replicate the dynamics of human movement and emotional expression – may create a simulation of an emotional state in the listener (Gridley and Hoff, 2006). Two brain regions, the posterior inferior frontal gyrus and the anterior insula, are commonly activated during musically evoked emotional states (Koelsch and Siebel, 2005; Menon and Levitin, 2005; Koelsch, 2010). Molnar-Szakacs and Overy (2006) propose that these structures are part of the simulation mechanism by which music communicates emotion to the listener. We hear the music as articulating dynamics akin to human emotional expressions and we (involuntarily) create a motor representation of that emotion within ourselves. The dynamics of musical gestures in these cases integrate with the listener’s relevant neural mechanisms, giving rise to autonomic and somatic responses that generate associated emotional responses, both physiological and phenomenological (Overy and Molnar-Szakacs, 2009).

At the behavioral level, music, as we have seen, solicits different forms of gestural and postural entrainment – overt physical expressions (e.g., facial expressions when listening to happy music) that induce the felt experience of emotions (cf. Ekman et al., 1983; Niedenthal, 2007). There is a great deal of empirical evidence indicating a reciprocal relation between an emotional experience and its behavioral expression. Simply adopting emotion-specific facial expressions or postures, for example, is often sufficient to bring about the associated experience (see Laird, 2007 for an overview). What is relevant for our purposes is that musical affordances scaffold our access to these emotions by quite literally pulling these emotion-inducing responses out of us (cf. Burger et al., 2013). But music’s impact does not stop there. By continuing to provide ongoing feedback, music serves as a real-time regulator: the temporal structure and periodic modulations of the music (melody, rhythm, volume, intensity, etc.) in its unfolding regulates the form that our physical expressions take (cf. Windsor and de Bézenac, 2012).^15^ In short, the music shapes our reactive behavior in a fine-grained way. Musical input (in the form of dynamic musical gestures) gives expressive shape to our reactive behavior, much the way that ongoing input from a dance partner extends, transforms – and in an important sense, *completes* – the shape of our *own* partner-dependent responses. The overt physiological and postural changes these musical gestures induce – and which arise via the auditory-motor integration characteristic of our perceptual engagement with these affordances – thus provide afferent feedback enhancing and transforming our affective state. When the music lifts, so do our expressive responses and the emotional states they induce; when it falls, so do we (cf. Witvliet and Vrana, 1996; Lundqvist et al., 2009). In manipulating our behavioral responses via patterns of entrainment, music thus manipulates our emotional experience by assuming a critical regulative function. We let music do some of the emotional work for us. And to return to an earlier point, this offloading is, I propose, a central part of the pleasurable “letting go” experience we tend associate with episodes deep listening: heightened experiences of music listening in which we feel as though we are experientially consumed by, and somehow taken up into, the music as it unfolds around us and leads us on a sonic and affective journey (Krueger, 2009, 2011b; cf. Bicknell, 2009; Gabrielsson and Bradbury, 2011). Within these experiences, we become acutely aware of the way that “musical agency” (Fritz et al., 2013) profoundly augments, extends, and organizes our own affective resources.

In sum, I suggest that, as with the performer–instrument relation, listener and music similarly form an integrated system (cf. Reybrouck, 2012). Within this system, the listener uses music (via musical affordances) as a kind of “esthetic technology” (DeNora, 2000) for regulating and transforming their behavior, attention, and emotion. In this sense, then, can music – when integrated with an appropriately responsive listener – function as an external cognitive and affective resource. Via processes of synchronization and bodily entrainment, music takes over some of the regulatory functions normally associated with subject-centered endogenous processes. When we attentively engage with music, we offload this cognitive function onto the music, much the way we do various memory functions onto environmental artifacts like notebooks and calendar apps in smartphones – and in so doing, enhance our cognitive performance. Music similarly affords this sort of externally driven regulation.

### MUSICKING AND NOVEL EMOTIONAL EXPERIENCES

Musical offloading potentially expands our affective repertoire and open ups new forms of experience. In adults, musical affordances enhance the functionality of endogenous process by providing additional regulative properties – properties which the listener exploits to access a more nuanced means of emotional refinement, attention, and expression than, say, the relatively coarse-grained possibilities offered by facial expressions, gestures, or postural adjustments (cf. Niedenthal, 2007). This nuance is reflected in how we tend to speak about music and its ongoing impact on the listener: its affective power, vitality, and seemingly infinite ability to convey a range of subtle feelings and expressions. Musical expressions of emotions can have, for instance, increased *complexity*, *temporal range*, *subtlety*, and *force* in contrast to their non-musical counterparts (Cochrane, 2008, p. 338). These properties help to explain why, when listening attentively to a piece of music, we often feel as though we have temporarily accessed a realm of feeling and expression that somehow goes beyond that of our everyday non-musical life; it also helps explain why musical expressions of emotion can seem simultaneously familiar and alien.^16^ When we integrate with music via musical affordances, we thus gain access to this expanded expressive palette, much the way that dancing with a highly skilled partner provides ongoing feedback that, for a time, at least, elevates one’s own dancing. As a result, we are able to temporarily access musically scaffolded forms of experience and expression that we cannot access outside of this music-listener system.

This is vividly demonstrated by looking at the narratives of those living with Moebius syndrome, a congenital form of bilateral facial paralysis (Cole, 1998; Briegel, 2006; Cole and Spalding, 2009). Since people with Moebius syndrome have no facial animation, they are unable to facially articulate emotions. They often report a diminishment or loss of emotion and affect that they feel results from their lack of facial expressivity (Cole, 2009). For example, one individual with Moebius writes that, “I think there’s a lot of dissociation [from emotional experience]. But I think I get trapped in my mind or my head. I sort of *think* happy or I *think* sad, not really saying or recognizing actually feeling happy or feeling sad⋯I have to say this thought is a happy thought and therefore I am happy”; another tells us that, “I did not express emotion. I am not sure I felt emotion, as a defined concept⋯I don’t think I was happy, or even had the concept of happiness as a child” (Cole, 2009, pp. 353–354).

These narratives appear to lend support to the previously discussed link between emotional experience and its behavioral expression (Laird, 2007; Niedenthal, 2007). But they are also interesting in virtue of what they tell us about the various *compensatory strategies* people with Moebius syndrome adopt (Bogart et al., 2012; Bogart et al., in press; Krueger and Michael, 2012). Since people with Moebius syndrome cannot use their face to express emotion, they often utilize other resources and strategies: modulating their tone of voice, intentionally speaking in a loud and clear voice, speaking candidly about their feelings, using humor, exaggerated gestures, touching, or employing various props or articles of clothing (e.g., a smiley face pin on the a jacket lapel; Bogart et al., 2012). These strategies involve recruiting compensatory strategies for expressing emotions to others. But people with Moebius syndrome also utilize compensatory strategies to *experience* emotion – and a common feature of their narratives is their reliance upon art and music in this regard (2009). The experience of both performing and listening to music seems in particular to be a means of accessing certain emotional and affective experiences – or at least emotional and affective experiences of a certain phenomenal *quality* or *intensity* – that, in virtue of their facial paralysis, they cannot otherwise access.^17^ One way to understand music’s efficacy in helping people with Moebius syndrome recalibrate their emotional phenomenology is, once again, to appeal to emotional offloading. People with Moebius syndrome offload certain regulatory and expressive functions onto the music – regulatory and expressive functions which they lack – and in so doing allow the music to scaffold experiences of a kind and degree that would otherwise remain inaccessible. Musical agency provides surrogate regulative functionality.

For all listeners, music can also scaffold our access to novel emotional and affective experiences by shaping our perception and experience of time. We tend to experience time as passing more slowly during sustained engagement with emotionally compelling music (Kellaris and Kent, 1992; Schäfer et al., 2013). This expanded sense of temporal presence offers additional regulative functionality. It allows us to attend more carefully, and in unaccustomed ways, to the development and modulation of our music-enhanced emotions. As an esthetic technology, music augments emotional expression *and* exploration. Again, when we engage with music, we potentially access an expanded horizon of regulative possibilities that bring with them a richer palate of emotional expression and experience than we could access purely by appealing to our own internal (i.e., non-musically enhanced) resources.

The regulatory functionality that musical affordances provide can also have a profound impact on the cognitive development and performance of children and young infants. In the case of young listeners, musical engagement can augment cognitive processes and emotional experiences that far exceed their current level of endogenous development. For example, background music has been shown to assist students with various developmental and learning disabilities by helping them to regulate their emotions, enhance motor coordination, and organize the attention needed to sustain task focus (e.g., Cripe, 1986; Hallam and Price, 1998; Savan, 1999).

More striking, however, is the impact that music has on very young perceivers (neonates and preterm infants) in terms of giving them access to elevated cognitive and affective competence. Neonates lack the neurobiological resources needed to self-regulate endogenous control of attention, emotion, and behavior (for details, see Rothbart, 1989; Gopnik, 2009, pp. 106–123). Accordingly, because their attention is largely exogenous (i.e., world-determined) at this stage of their development, they are extremely vulnerable to environmental perturbations. The input of caregivers thus becomes extremely important in managing their attention and stabilizing their affect. Caregivers assist infants here by employing a range of different physical strategies (exaggerated movements, gestures, and facial expressions; manipulating gaze, body orientation; “infant-directed” speech consisting of raised pitch, slowed tempo, elongated vowels, and slow pitch contours with large frequency ranges) designed to actively modulate the attentional and affective character of early interactions in a way that is vital for the infant’s social-cognitive development (Stern, 1985; Tronick, 2005; cf. Krueger, 2013b). However, musical affordances can, as we saw previously, integrate with the infant’s native interest in and responsiveness to music and provide surrogate endogenous function, organizing attention, behavior, and affect in a way that exceeds their current level of development.

For example, not only do infants display an evaluative preference for consonant over dissonant music, in that the former guides their attention toward it while the latter repels it (Trainor and Heinmiller, 1998, p. 83). Additionally, they latch onto affordances specific to the former in order to regulate their internal states and bring about a more inquisitive and emotionally balanced state in relation to their environment (Zentner and Kagan, 1998). In Zentner and Kagan’s study, the infants actively engaging with consonant music fretted less, exhibited slower and more controlled motor activity (i.e., they were less fidgety while absorbed in the music) and vocalized more (i.e., expressed interest in the music) than when listening to dissonant music or non-musical sounds. In short, they exhibited significantly enhanced behavioral, attentional, and emotional organization within a specifically musical context. Haslbeck (2004) similarly found that pre-term infants entrained bodily movements (sucking, tongue and mouth protrusions, eye opening and closing), respiratory patterns, and alerting responses with consonant music (in this case, lullabies) and, in so doing, exhibited a heightened regulatory competence specific to the music therapeutic context. In these cases, the music does more than provide sensory input to be processed by the infant’s internal resources. Rather, it assumes an external regulative function that determines the infant’s behavior and affective responses. The distributed listener–music dynamics of this ongoing, mutually modulatory exchange are where the infant (for a time, at least) is able to realize novel musically enhanced cognitive and emotional feats.

## CONCLUSION

I have argued for a picture of the musically extended mind. I sketched a picture of “musical affordances” and argued that, in some circumstances, musical dynamics provided ongoing resources and feedback that enhance the functional complexity of various motor, attentional, and regulative capacities responsible for generating and sustaining emotional experience. A cascade of neural and behavioral entrainment responses are what secure the sort of sustained integration with musical affordances needed to bring about music-specific regulatory processes. In these cases, I claimed, specifying the nature of the relevant processes – and the emotions they give rise to – requires that we look beyond the boundary of the individual.

I do not claim to have conclusively defended the existence of a musically extended emotional mind. More work would need to be done – for example, to specify why an *extended* way of thinking about musically induced emotions is explanatorily superior to adopting a more conservative *situated* approach (cf. Rupert, 2004; Sprevak, 2010). I would also need to explicitly address the much-discussed coupling-constitution objection (Adams and Aizawa, 2001, 2008): the objection that, just because a given cognitive process is *causally* dependent upon an environmental process, it does not follow that the environmental processes thereby becomes a *constitutive part* of the causal process. I have responded to this objection elsewhere (Krueger, 2012). For what it is worth, I do not find this objection as formidable as is often assumed, in part because it strikes me as dangerously close to being question-begging (see, e.g., Hurley, 2010; Ross and Ladyman, 2010; Kagan and Lassiter, 2013). But that is a discussion for another time. My point is simply that, if we take seriously the possibility that certain environmental resources can scaffold the emergence of extended emotions – as an increasing number of philosophers and cognitive scientists are inclined to do – we ought to take seriously the possibility that music is a particularly powerful example an emotion-extending resource.

Few would dispute that music plays a powerful role in our everyday lives. Its ubiquity and enduring popularity testifies to its ongoing impact; without music, our lives would, indeed, be greatly impoverished. But if something like the above story is true, the loss of music would not only diminish their esthetic quality but, additionally, our capacities as emotional agents. Thankfully, we still have music – and as such, a musically extended mind.

## Conflict of Interest Statement

The author declares that the research was conducted in the absence of any commercial or financial relationships that could be construed as a potential conflict of interest.

## Footnotes

1 In what follows, I use “emotions,” “affect,” and “feelings” interchangeably. A further clarificatory point: I am not suggesting that we always use music to generate powerful feelings. Sometimes our felt responses to music are mild – and sometimes, non-existent. Nevertheless, musical experience plays an important role in nearly all cultures in part because of its potential for eliciting strong individual and collective affective responses (Juslin and Laukka, 2004; see also Becker, 2004; Bicknell, 2009; Gabrielsson and Bradbury, 2011). Moreover, it is telling that, as far as we know, caregivers in all cultures regularly sing to their infants as a means of emotion regulation (Trehub and Nakata, 2001).

2 We have been musicking for quite a long time. The discovery of musical instruments over 35,000 years old suggest that well-established musical practices and traditions were a central part of human life far earlier than first thought (Conard et al., 2009).

3 Of course, not all funeral ceremonies are sombre affairs – such as the often raucous, celebratory tone of African–American funerals in the Southern Baptist tradition in the United States. While these services may begin with mournful singing and prayer, they often end on an extremely joyful note: an energetic celebration of the deceased’s reunion with Christ. Even in these cases, however, the music shapes the atmosphere of the service as it transitions from grief to joyful celebration.

4 An instrumentalist perspective on musical engagement cuts against the more cerebral grain of much music cognition research, according to which the primary role of the listener is to passively contemplate the piece and, in so doing, attempt to discern its inherent meaning (Small, 1998, p. 6). Sloboda (2005, p. 319) dismissively terms this the “pharmaceutical model” of music consumption. It presupposes that listeners passively receive and respond to musical stimuli in virtue of determinate relations between musical structures and features of our psychology, analogous to the way that taking specific drugs has determinate neurochemical and psychological effects (see Huron, 2006 for an ingenious example of such a cerebral approach). An instrumentalist approach, to the contrary, stresses the active role the listener plays in shaping both how they listen to music and what they hear when they do.

5 I am here parting ways with Gibson, who was not particularly interested in the phenomenology of affordances (see Dreyfus and Kelly, 2007; see also Sanders, 1999; Michaels, 2003).

6 An ecological approach to music performance can also be highly illuminating (see, e.g., Davidson and Good, 2002; Borgo, 2005), but I will here focus on the experience of music listening.

7 Although, strictly speaking, our engagement with music is not really one-way since, as we will see, the experiential character of the music-as-perceived – as well as the sort of reactive behavior we experience the music as affording – is shaped by the form of our “advancing behaviour.”

8 The allure of music as a resource for entraining is not necessarily confined to human perceivers (see Patel et al., 2009).

9 In the embodied cognition literature, “offloading” refers to the manner by which we overcome cognitive limitations (e.g., limits on attention and working memory) by exploiting our environment in a strategic way to reduce our cognitive workload. For example, we post sticky notes on the side of our computer monitor or place a box full of cans bound for the recycling bin by the front door as a memory prompt. In these cases, we offload information onto the environment for later recall so that we can conserve our resources for more immediate tasks (cf. Kirsh, 1995; Wilson, 2002).

10 Musical features alone do not determine the “fit” of entrainment responses. Cultural norms also play an important role here (Cross, 2001; Hannon and Trainor, 2007).

11 For more on the idea of experientially inhabiting musical spaces, (see Krueger, 2009, 2011b).

12 I invite the reader sceptical of this musical vs. non-musical contrast to compare the experience of entraining with the steady pulsing of a car alarm, say, or ticking of a clock with that of any random pop music hit. We can, of course, entrain with non-musical sounds. But I am confident that, for most of us – congenital amusiacs excluded (Ayotte et al., 2002) – the experiential character of entraining with music is to experience it as potentiating a range of reactive responses that far exceeds those of a car alarm or clock. Again, this potentiating character is part of what specifies music’s uniquely potent affective allure. Of course, pieces within some musical genres (e.g., avant-garde jazz, noise-based avant-garde rock, minimal electronic ambient, etc.) lack a well-articulated rhythmic structure and thus may initially seem to present fewer entrainment possibilities than others (e.g., radio-friendly pop music). A seasoned listener, however, may eventually discern subtle variations in texture, tempo, or modulations of volume and intensity that suggest creative entrainment possibilities; this possibility of perpetual discovery is surely one of the reasons why the same piece of music remains sonically compelling across multiple listens. However, we must also concede that there may be instances where certain pieces present no entrainment possibilities whatsoever – but we are nevertheless still inclined to speak of them as music. I have no intention of placing a priori constraints on what counts as music by suggesting that all music, insofar as it is to count as “music,” must of necessity provide entrainment possibilities. I am grateful for two reviewers pressing me on this point.

13 Although in virtue of its dynamic quality, music may represent or mirror something analogous to the way that we bodily express emotions in the kinematics of our gait, posture, gestures, facial expressions, vocalisations, etc. (cf. Molnar-Szakacs and Overy, 2006; Juslin and Västfjäll, 2008). But this claim is distinct from saying that music represents the emotion itself. I return briefly to this idea below.

14 Clark himself appeals to musical examples to support this idea. He tells us that “continuous reciprocal causation [is not] a rare or exceptional case in human problem solving. The players in a jazz trio, when improvising, are immersed in just such a web of causal complexity. Each member’s playing is continually responsive to the others’ and at the same time exerts its own modulatory force” (Clark, 1997, p. 165). It should be noted, however, that the continuous reciprocal causation found in music listening has a somewhat different structure than prototypical cases of extended cognition. Unlike Otto recording new information in his notebook or a jazz musician actively modulating her partner’s performance, there is no analogous environmental manipulation; rather, the relevant changes take place in the listener (i.e., their experience of the music as affording different forms of entrainment and emotion regulation). Nevertheless, it is not clear that this difference threatens the prospects for genuine integration (and subsequent extension). For, what is required, I suggest, is not necessarily the presence of a genuine material change (i.e., of the music considered as a thing in the world) but rather an informational change – that is, the way that affectively salient resources in the music-as-perceived are detected and acted on, and what sort of downstream modulatory impact they have on shaping the listener’s further reactive behaviour, which in turn shapes how different affordances are detected, etc. Like the information in Otto’s notebook, this information is “stored” in an external resource; and it is similarly poised to provide real-time cognition-enhancing, behaviour-guiding resources for a suitably attuned and responsive listener (cf. Clark, 2010). This is a complicated issue, however, and requires much more discussion than I can here give it. I am very grateful to a reviewer for pressing for further clarity on this point.

15 Recall the earlier discussion of music and strenuous activity, as well as the discussion of how the acoustic structure of specific musical styles (Salsa, Tango, Post-Rock, Electronic, etc.) affords complementary patterns of entrainment with their own unique temporal signatures and affective synchrony.

16 Similar intuitions likely led Schopenhauer to declare music alone among the arts uniquely capable of providing access to the essence of the world in itself (i.e., the world as pure “will”; Schopenhauer, 2011).

17 This is not to suggest that people with Moebius syndrome live utterly impoverished emotional lives. Far from it. The point, rather, is that certain emotional experiences seems to be diminished in virtue of their loss of facial expressivity. But even here, these experiences can be compensated for by adopting various alternative strategies available both to the person with Moebius as well as to those they interact with (Bogart et al., 2012).
